# Testosterone deficiency reduces cardiac hypertrophy in a rat model of severe volume overload

**DOI:** 10.14814/phy2.14088

**Published:** 2019-05-03

**Authors:** Catherine Beaumont, Élisabeth Walsh‐Wilkinson, Marie‐Claude Drolet, Élise Roussel, Nicolas Melançon, Émile Fortier, Geneviève Harpin, Jonathan Beaudoin, Marie Arsenault, Jacques Couet

**Affiliations:** ^1^ Groupe de recherche sur les valvulopathies Centre de Recherche Institut universitaire de cardiologie et de pneumologie de Québec Université Laval Quebec City Canada

**Keywords:** Androgens, aortic regurgitation, cardiac hypertrophy, left ventricle, rat, testosterone

## Abstract

The aim of the study was to characterize if the development of cardiac hypertrophy (CH) caused by severe left ventricle (LV) volume overload (VO) from chronic aortic valve regurgitation (AR) in male rats was influenced by androgens. We studied Wistar rats with/without orchiectomy (Ocx) either sham‐operated (S) or with severe AR for 26 weeks. Loss of testosterone induced by Ocx decreased general body growth. Cardiac hypertrophy resulting from AR was relatively more important in intact (non‐Ocx) animals than in Ocx ones compared to their respective S group (60% vs. 40%; *P* = 0.019). The intact AR group had more LV dilation, end‐diastolic LV diameter being increased by 37% over S group and by 17% in AROcx rats (*P* < 0.0001). Fractional shortening (an index of systolic function) decreased only by 15% in AROcx compared to 26% for intact AR animals (*P* = 0.029). Changes in LV gene expression resulting from CH were more marked in intact rats than in AROcx animals, especially for genes linked to extracellular matrix remodeling and energy metabolism. The ratio of hydroxyacyl‐Coenzyme A dehydrogenase activity over hexokinase activity, an index of the shift of myocardial substrate use toward glucose from the preferred fatty acids, was significantly decreased in the AR group but not in AROcx. Finally, pJnk2 LV protein content was more abundant in AR than in AROcx rats, indicating decreased activation of this stress pathway in the absence of androgens. In summary, testosterone deficiency in rats with severe LV VO resulted in less CH and a normalization of the LV gene expression profile.

## Introduction

Cardiovascular diseases are among the leading causes of mortality in both men and women (Roth et al. 2017). Men are usually affected earlier in life and progression is often faster than for women (Leinwand 2003). On the other hand, women usually suffer from heart diseases later in life, usually after menopause (Roger et al. 2012). Cardiac hypertrophy (CH), an independent cause of morbidity and mortality from heart diseases also displays a sex dimorphism in both patients and in pre‐clinical models (Blenck et al. 2016).

CH develops in response to a recurrent or chronic significant hemodynamic stress. A pathological stimulus will lead to maladaptive CH and eventually if left untreated, to heart failure (HF). Significant pressure overload (e.g., hypertension, aortic valve stenosis) or volume overload (e.g., aortic or mitral valve regurgitation) will induce left ventricle (LV) remodeling characterized by changes in wall thickness, chamber diameter, extracellular matrix organization, gene transcription, cell signaling, and energy metabolism (Grossman et al. 1975; Witt et al. 2008; Champetier et al. 2009; Lopaschuk et al. 2010).

We recently observed that in a rat model of chronic LV volume overload (VO) caused by severe aortic valve regurgitation (AR), females developed as much if not more CH than males. However, male showed more LV dilation but lost more contractile function than females (Beaumont et al. 2017). In another rat VO model (aorto‐caval fistula), a faster progression toward HF was observed in males and resulted in poorer survival (Gardner et al. 2002; Dent et al. 2010). At the cellular and molecular levels, we also observed that male AR rat showed an important downregulation of many LV fatty acid oxidation (FAO) genes and an upregulation of glucose metabolism genes, whereas this energy metabolism switch characteristic of pathological CH did not happen in females (Beaumont et al. 2017). Sex steroids have a potent effect on differentiation and may explain a large part of the sex dimorphism observed in CH (Mahmoodzadeh et al. 2012).

Estrogens have been widely studied in the field of cardiovascular research, especially because of the lower incidence of cardiovascular diseases in premenopausal women compared to men (Blenck et al. 2016). Effects of androgens on heart physiology are less well understood. It is not clear if androgens are protective or deleterious for the cardiovascular system. Studies have shown that low testosterone levels predispose men to a higher risk of cardiovascular disease. On the other hand, testosterone can increase functional capacity and cardiac output in men with chronic HF (Pugh et al. 2003; Malkin et al. 2006). Loss of testosterone has recently been shown to increase development of diastolic dysfunction in aging male mice (Ayaz et al. 2019). Nevertheless, some studies associated testosterone replacement therapy with a higher risk of cardiac events (Xu et al. 2013). Cardiomyocytes treated with androgens (testosterone and/or dihydrotestosterone) show a hypertrophic response (Marsh et al. 1998) in agreement with their well‐known anabolic effect on muscle hypertrophy (Basualto‐Alarcón et al. 2013). The androgens effects on the development of pathological CH are thus still up for debate.

Here, we wanted to study if androgens would influence the development of eccentric left ventricle hypertrophy in the rat AR model. Considering that androgens are only produced by testes in rats (van Weerden et al. 1992), we studied the hearts of intact and orchiectomized animals (Ocx) with or without VO induced by AR. Our results suggest that androgens do influence cardiac remodeling in this VO model and have an impact on the heart response to this hemodynamic stress.

## Methods

### Animals

Forty‐four male Wistar rats (275–300 g) (Charles River, Saint‐Constant, QC, Canada) were studied. Twenty‐two of which were purchased after Ocx at age 8 weeks. Experimental groups were as followed: Sham‐operated (S; *n* = 9), Ocx sham‐operated (SOcx; *n* = 10), AR; (*n* = 13) and Ocx AR (AROcx; *n* = 12). AR was induced 2 weeks later at age 10 weeks as previously described by perforation of one or two aortic valve leaflets using a catheter via the right carotid and under echocardiographic guidance (Arsenault et al. 2002; Plante et al. 2003). Sham‐operated animals only had ligation of their right carotid. The protocol was approved by the Université Laval's Animal Protection Committee and followed the recommendations of the Canadian Council on Laboratory Animal Care.

### Echocardiography

An echocardiographic exam was performed 2 weeks after surgery to confirm AR severity and at the end of the protocol 26 weeks later as previously described (Plante et al. 2006; Lachance et al. 2009b; Arsenault et al. 2013). At the end of the protocol, the heart and the lungs were harvested and weighed. Heart chambers were dissected, weighted, and the LV was then quickly frozen in liquid nitrogen and kept at −80°C until further use. A piece of LV was kept in formalin and fixed in paraffin for histologic analysis.

### Gene expression analysis by RT‐PCR

LV gene expression was quantified for six animals per group by quantitative RT‐PCR as described elsewhere (Plante et al. 2008; Champetier et al. 2009). Briefly, LV RNA samples were diluted to 500 ng/*μ*L. One‐microliter of RNA (500 ng) was converted to cDNA using the QuantiTect^®^ Reverse Transcription kit (Qiagen), a procedure which included a genomic DNA elimination step. The cDNA obtained was further diluted 11‐fold with water prior to amplification (final concentration corresponding to 4.54 ng/*μ*L of initial RNA). Five‐microliter of diluted cDNA were amplified in duplicate by Q‐PCR in a Rotor‐Gene™ thermal cycler (Corbett Life Science. Sydney, Australia), using optimized specific primer pairs (Table S1) and SsoAdvanced Universal SYBR Green Supermix (Bio Rad, Hercules, CA). Each run included one tube with water only (no template control) and a series of three 10‐fold dilutions of a representative cDNA sample to check efficiency of the amplification reactions. In the case of intron‐less genes, minus‐RT control reactions were made on ten randomly chosen samples to ensure that genomic DNA did not yield significant amplification. The quantification of gene expression was based on the −2ΔΔCt method. Mean Ct values of duplicates for each gene of interest were subtracted from the mean Ct value (ΔCt) of the control “housekeeping” gene cyclophilin A. The difference in the mean ΔCts between groups of rats (ΔΔCt) allows the calculation of relative levels of induction/repression of genes of interest. The non‐preoptimized primers were for the 2,4‐dienoyl‐CoA reductase 1 gene (*Decr1*; 5′‐CAT TCC GTA TCT ACC CCA TTC AG‐3′ and 5′‐GCT ATC ACT ACG ATC TAT GCT GAG‐3′; 120 pb transcript), enoyl‐CoA hydratase, short chain 1 (*EchS1*; 5′‐GCT TTC AGG GTG TCT TGA TTT G‐3′ and 5′‐GAG CTA TGC ACT GCA GAT AGT‐3′; 95 pb transcript), natriuretic peptide precursor type A (*Nppa*; 5′‐GCA GAT TTG GCT GTT ATC TTC G‐3′ and 5′‐GGT AGG ATT GAC AGG ATT GGA‐3′; 79 pb transcript), natriuretic peptide precursor type B (*Nppb*; 5′‐GTC TCC CTA AAA CAA CCT CAG C‐3′ and 5′‐CGA AAT TCC AAG ATG GCA CAT AG‐3′; 108 pb transcript), and Cyclophilin A (*Ppia*; 5′‐GCA GAC AAA GTT CCA AAG ACA G‐3′ and 5′‐CCA TTA TGG CGT GTG AAG TC‐3′; 140 pb transcript).

### Enzymatic activity

Enzymatic activities (*V*_max_) for hexokinase (HK), hydroxyacyl‐Coenzyme A dehydrogenase (HADH), succinate dehydrogenase (SDH), citrate synthase (CS), and creatine kinase (CK) were determined as previously described (Bouchard‐Thomassin et al. 2011; Dhahri et al. 2014).

For the beta‐hydroxybutyrate dehydrogenase (BDH) enzyme assay, LV tissue was homogenized with 40 volumes of an extracting solution. The extracting solution was as follows: triethanolamine (50 mmol/L; pH 7.5), EDTA (1 mmol/L), MgCl_2_ (2 mmol/L), and *β*‐mercaptoethanol (30 mmol/L). The homogenate was then sonicated. NADH formation was monitored by UV spectroscopy at 340 nm as an indication of BDH activity. The samples were tested in duplicates.

### Immunoblotting

Protein content was estimated by Western blotting as described previously (Belanger et al. 2003; Plante et al. 2004). Antibodies were diluted 1:1000 in a TSB‐T solution with 5% bovine serum albumin (BSA). Phospho‐ERK1/2, phospho‐JNK1/2, phospho‐p38, and phospho‐GSK3 were all obtained from Cell Signaling Technologies (Danvers MA) whereas ERK1/2 antibody was from Millipore, (Etobikoke, ON, Canada) and p38 from Santa Cruz Biologicals, (Santa Cruz CA). GSK3 (Santa Cruz Biologicals) and phospho‐FAK (Cell Signaling Technologies) were diluted 1:500 in TSB‐T solution with 5% BSA. Antibodies diluted in a TBS‐T solution with 5% milk were phospho‐Akt Thr308 (1:500, Cell Signaling Technologies), Akt (1:1000, Cell Signaling Technologies), JNK2 (1:1000, Santa Cruz), PTEN (1:1000, Cell Signaling Technologies), and FAK (1:1000, Santa Cruz). Antibodies diluted in TBS‐T only were phospho‐Akt Ser473 (1:2000, Cell Signaling Technologies), phospho‐S6 (1:2000, Cell Signaling Technologies), S6 (1:2000, Cell Signaling Technologies), phospho‐PKD (1:1000, Cell Signaling Technologies), and PKD (1:500, Abcam, Toronto, ON, Canada). All antibodies were from rabbits, except for JNK2 and Akt, which were from mice. The appropriate second antibody was diluted 1:2000 in TBS‐T with 5% milk. Western Lightning Plus ECL (Perkin‐Elmer, Woodbridge, ON, Canada) and Clarity Max ECL (Bio‐Rad) were used for membrane revelation with the Gel Doc system and Image Lab software (Bio‐Rad). For normalization, the “Total lane method” was used and adjustments were made according to pre‐blocking membrane exposure and to a standard pool.

### Histology

Picrosirius red staining was done in order to measure mid‐LV sections size and fibrosis content (*n* = 6/group). LV pieces fixed in paraffin were cut and deparaffinized. Tissue sections were then rehydrated and then colored for 1 h in 0.1% Sirius red diluted in saturated picric acid. Sections were then washed in 1% acetic acid, dehydrated three times in 100% ethanol for 5 min and finally washed in xylene. A picture of each mid‐LV section was acquired with a Zeiss optical microscope and Zen software (Germany) with a size of 280 equal area tiles.

### Statistical analysis

Results are presented as the mean ± the standard error of the mean (SEM). Two‐way ANOVA was performed and Holm‐Sidak post‐test was used for comparison between the groups (Graph Pad Prism 7.04, San Diego, CA). A Student's *t*‐test was used when only two groups were compared. Survival was analyzed by standard Kaplan–Meier analysis with log‐rank test. A *P* < 0.05 was considered significant.

## Results

**Figure 1:**
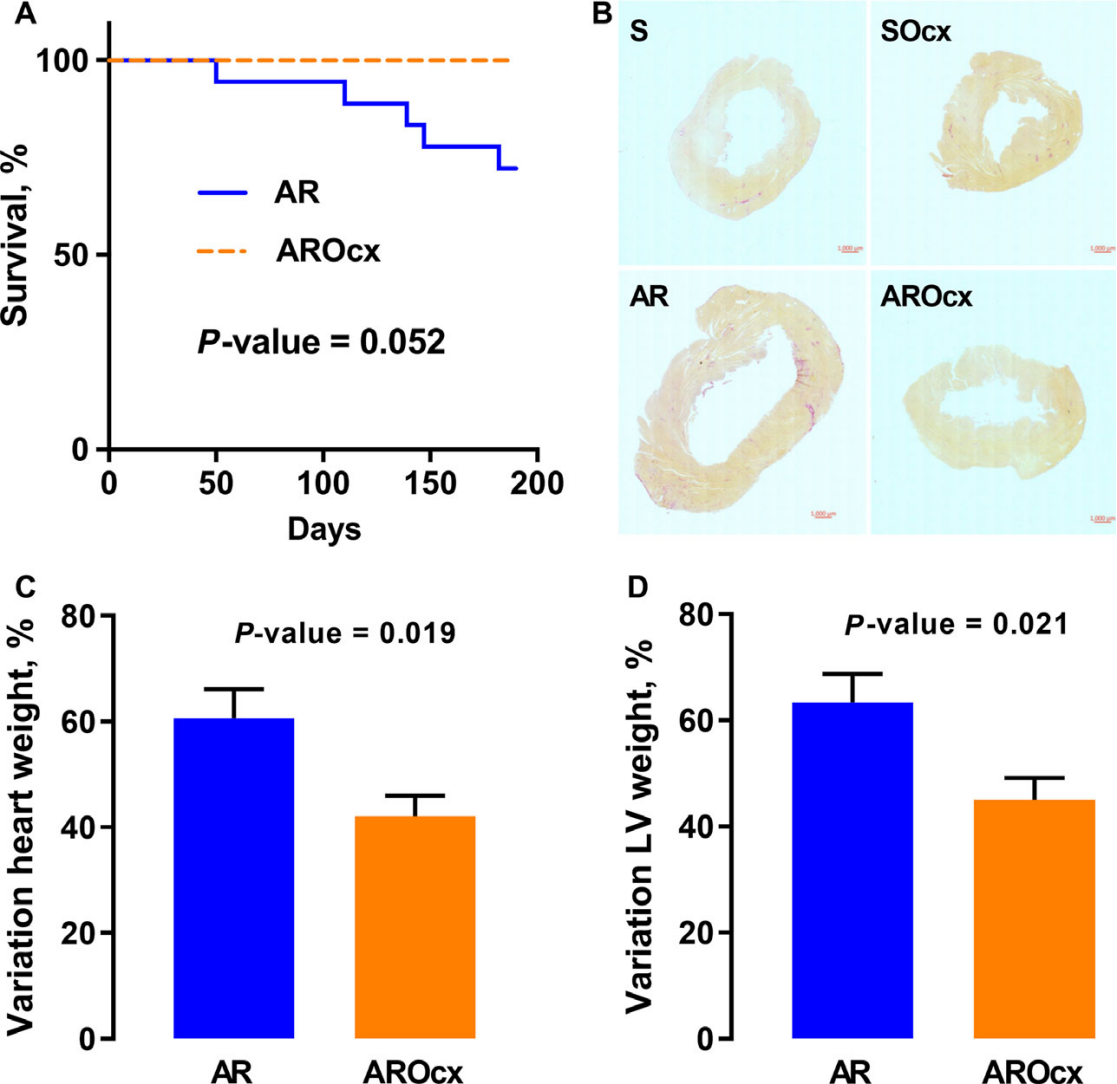
Survival of animals with AR during the protocol (A), mid LV histological short‐axis sections (B). Variation of heart weight (C) and LV weight (D) after tissue harvesting (*n* = 8–10/gr). For (C) and (D), the measured parameters of animals with AR were reported on those of their respective sham group to obtain the % of variation. For (A), *P*‐value from Kaplan‐Meier Log‐rank test. For C and D, Student's *t*‐test.

Eight out of 13 AR rats were alive at the end of protocol. All animals in the other groups survived the duration of the protocol. Difference in survival between AR and AROcx groups was close to statistical significance (*P* = 0.052) (Fig. 1A). Ocx animals had a significantly lower body weight than intact animals (*P* < 0.0001). There were no significant changes in lungs weight (a marker of heart failure) between the S and the AR and between the SOcx and the AROcx groups (Table 1).

**Table 1:** Animal characteristics at the end of the protocol

Parameters	S (*n* = 9)	AR (*n* = 8)	SOcx (*n* = 10)	AROcx (*n* = 9)
Body weight, g	820 ± 25	751 ± 20¶	562 ± 17^T^	573 ± 14^T^
Tibia, mm	60.5 ± 0.2	60.7 ± 0.4	56.0 ± 0.2^T^	56.1 ± 0.3^T^
Heart, mg	1549 ± 27	2487 ± 85¶	1122 ± 24^T^	1594 ± 43¶T
Indexed Heart, mg/g	1.90 ± 0.05	3.32 ± 0.10¶	2.01 ± 0.05	2.82 ± 0.10¶T
LV, mg	1182 ± 17	1956 ± 64¶	861 ± 20^T^	1249 ± 36¶T
Indexed LV, mg/g	1.45 ± 0.04	2.62 ± 0.08¶	1.54 ± 0.04	2.21 ± 0.09¶T
RV, mg	277 ± 14	366 ± 24¶	187 ± 8^T^	252 ± 17¶T
Indexed RV, mg/g	0.34 ± 0.02	0.49 ± 0.03¶	0.33 ± 0.01	0.44 ± 0.03¶
Lungs, g	3.2 ± 0.2	3.4 ± 0.2	2.2 ± 0.2^T^	2.3 ± 0.2^T^

S, sham control group; SOcx, sham orchiectomized group; AR, aortic regurgitation control group; AROcx, AR orchiectomized group; LV, left ventricle; RV, right ventricle. Values are expressed as the mean ± SEM. Group comparisons were made with Two‐way ANOVA and Holm‐Sidak post‐test. ^¶^ *P* < 0.05 versus the respective sham group and ^T^ *P* < 0.05 versus the respective intact (non‐Ocx) group.

### Cardiac hypertrophy

LV volume overload from AR caused a significant gain in cardiac mass for both intact and Ocx animals (*P* < 0.0001) (Fig. 1C). This gain was larger for the AR group compared to AROcx (60% vs. 40%, respectively; *P* = 0.019). AR severity was similar between the groups (Table 2). Hypertrophy from AR was more important in the LV compared to the RV and the LV gain of mass compared to respective S group was more important in intact AR animals than in AROcx (LV: 66%, RV: 45% for AR compared to LV: 45%, RV: 32% for AROcx, respectively) (Table 1 and Fig. 1D). The important LV hypertrophic response in AR animals was also apparent on a left mid‐ventricular section. The AROcx LV tended to be similar to S animals (Fig. 1B). We did not recorded significant changes in the content of interstitial myocardial fibrosis between the groups (not illustrated).

**Table 2:** Echocardiographic and hemodynamic parameters at the end of the protocol

Parameters	S (*n* = 9)	AR (*n* = 8)	SOcx (*n* = 10)	AROcx (*n* = 9)
EDD, mm	9.41 ± 0.08	12.9 ± 0.2¶	8.8 ± 0.1^T^	10.2 ± 0.2¶T
ESD, mm	4.7 ± 0.1	8.1 ± 0.3¶	4.6 ± 0.1	6.1 ± 0.3¶T
SW, mm	1.24 ± 0.04	1.83 ± 0.04¶	1.13 ± 0.02	1.27 ± 0.05¶T
RWT, unitless	0.263 ± 0.008	0.285 ± 0.005	0.257 ± 0.005	0.250 ± 0.008^T^
FS, %	50.1 ± 1.2	37.1 ± 1.6¶	47.4 ± 1.1	40.3 ± 1.6¶
HR, bpm	362 ± 8	345 ± 12	362 ± 8	384 ± 6
SV, *μ*L	399 ± 7	634 ± 33¶	364 ± 5	498 ± 22¶T
CO, mL/min	144 ± 4	217 ± 12¶	132 ± 4	187 ± 11¶
Aortic reg., %	N/A	85 ± 2	N/A	79 ± 3

EDD, end‐diastolic diameter; ESD, end‐systolic diameter; SW, septum wall thickness; RWT, relative wall thickness; FS, fractional shortening; HR, heart rate; SV, stroke volume; CO, cardiac output; bpm, beats per minute; Aortic reg., aortic regurgitation; N/A, not applicable. Values are expressed as the mean ± SEM. Group comparisons were made with Two‐way ANOVA and Holm‐Sidak post‐test. ^¶^ *P* < 0.05 versus the respective sham group and ^T^ *P* < 0.05 versus the respective intact group.

### Echocardiographic data

**Figure 2:**
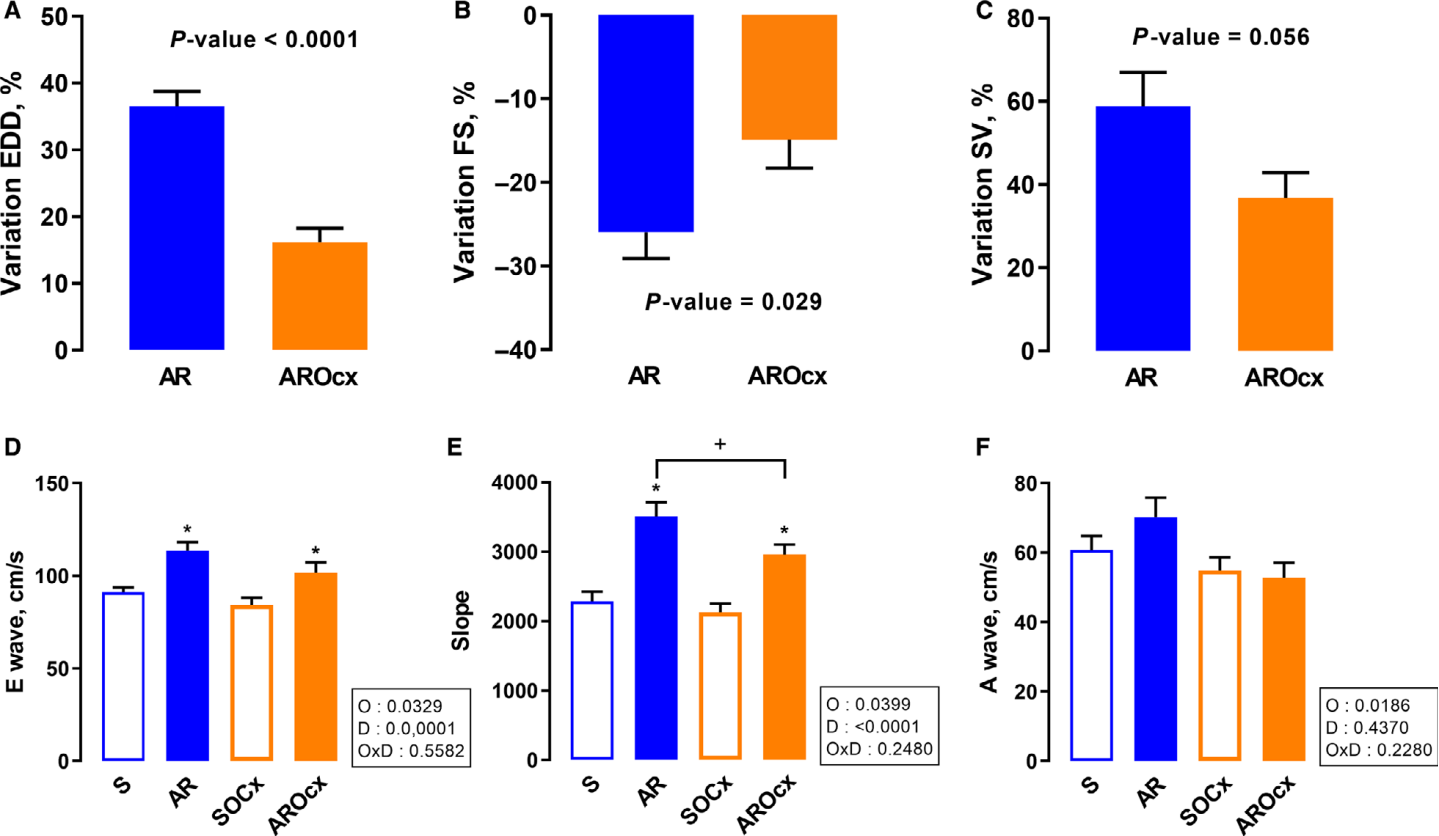
Echocardiographic parameters on LV diastolic diameter, systolic function and compliance evaluated at the end of the protocol (*n* = 9–12/gr). Variations of end‐diastolic diameter (A), of fractional shortening (FS) (B) and of stroke volume (C). Evaluation of the E wave (D), E‐wave slope (E) and A wave (F). For A–C, *P*‐value above the graph is from Student's *t*‐test. (D–F) Statistics in the boxes next to each graph are from two‐way ANOVA and Holm‐Sidak post‐test. *P*‐value of orchiectomy (O), disease (D) and interaction (OxD), *P < 0.05 versus their respective sham group and ^+^ *P* < 0.05 between the indicated groups.

LV dilation was significantly greater in AR than in AROcx rats as illustrated by the end‐diastolic (EDD) dimensions (Fig. 2A and Table 2). Inter‐ventricular septal wall thickness (SW) increased in AR compared to sham animals, but this increase was significantly greater in intact rats (*P* < 0.0001) (Table 2). A larger decrease in LV fractional shortening (FS) was observed in intact AR animals compared to AROcx (−26% vs. −15%, respectively) (Fig. 2B). As expected, LV stroke volume (SV) was significantly increased in AR and AROcx males. Again, there was a tendency toward a more important SV increase in intact AR than in AROcx male rats (*P* = 0.056) (Fig. 2C). Heart rates were similar between the groups (Table 2).

We measured diastolic flow parameters through the mitral valve. E wave, representing LV rapid filling, was significantly higher in AR animals (Fig. 2D). Likewise, the slope of the E wave was significantly steeper for AR rats. Again, these changes were more important for AR rats than for AROcx (Fig. 2E). The A wave was reduced in Ocx compared to intact rats (Fig. 2F). There was no significant difference in the E/A ratio between the groups (not shown).

### Markers of hypertrophy and extracellular matrix remodeling

**Figure 3:**
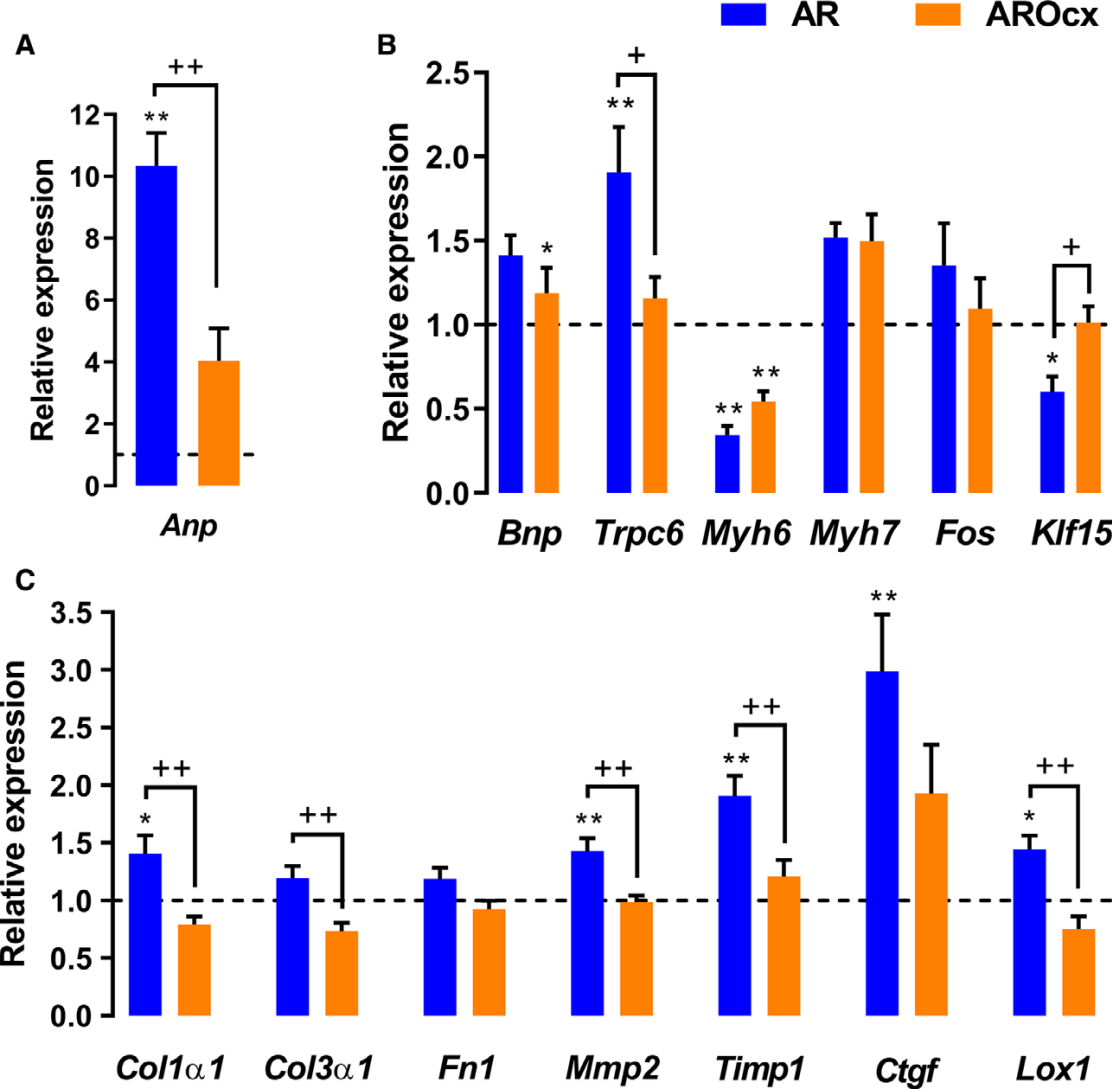
Evaluation by real‐time quantitative RT‐PCR of the LV mRNA levels of genes encoding for hypertrophy (A, B) and extracellular matrix remodeling (C) markers. The results are reported in arbitrary units (AU) as the mean ± SEM (*n* = 5–6/gr.). Messenger RNA levels of the S group were normalized to 1 and are represented by the dotted line. The blue column represents the AR animals while orange columns represent AROcx animals. *P < 0.05 or **P < 0.01 versus their respective sham group and ^+^ *P* < 0.05 or ^++^ *P* < 0.01 between the indicated groups.

AR caused an increase in LV *Anp* gene expression, this being significantly stronger in intact animals (Fig. 3A and B). *Bnp* expression was similarly increased in both AR groups. *Trpc6* expression was only increased in the intact AR group. As expected, *Myh6* gene expression was reduced with AR whereas *Myh7* expression tended to increase. *Klf15*, a transcription factor inhibiting the expression of pro‐hypertrophic genes (Leenders et al. 2012), was only downregulated in intact AR animals. Orchiectomy, itself, had a little influence on basal LV mRNA levels of the genes described above (Fig. S1).

We then analyzed gene expression of several extracellular matrix (ECM) remodeling markers. In intact AR animals, *Col1α1*,* Mmp2*,* Timp1*,* Ctgf*, and *Lox1* mRNA levels were significantly increased compared to sham‐operated rats (Fig. 3C). Gene expression for most of those genes in AROcx animals was similar to S controls. There was a tendency for most of these genes to have a lower expression in the LV of Ocx animals, this being significant for *Col1α1* and *Col3α*1 (Fig. S1).

### LV energy metabolism and markers

**Figure 4:**
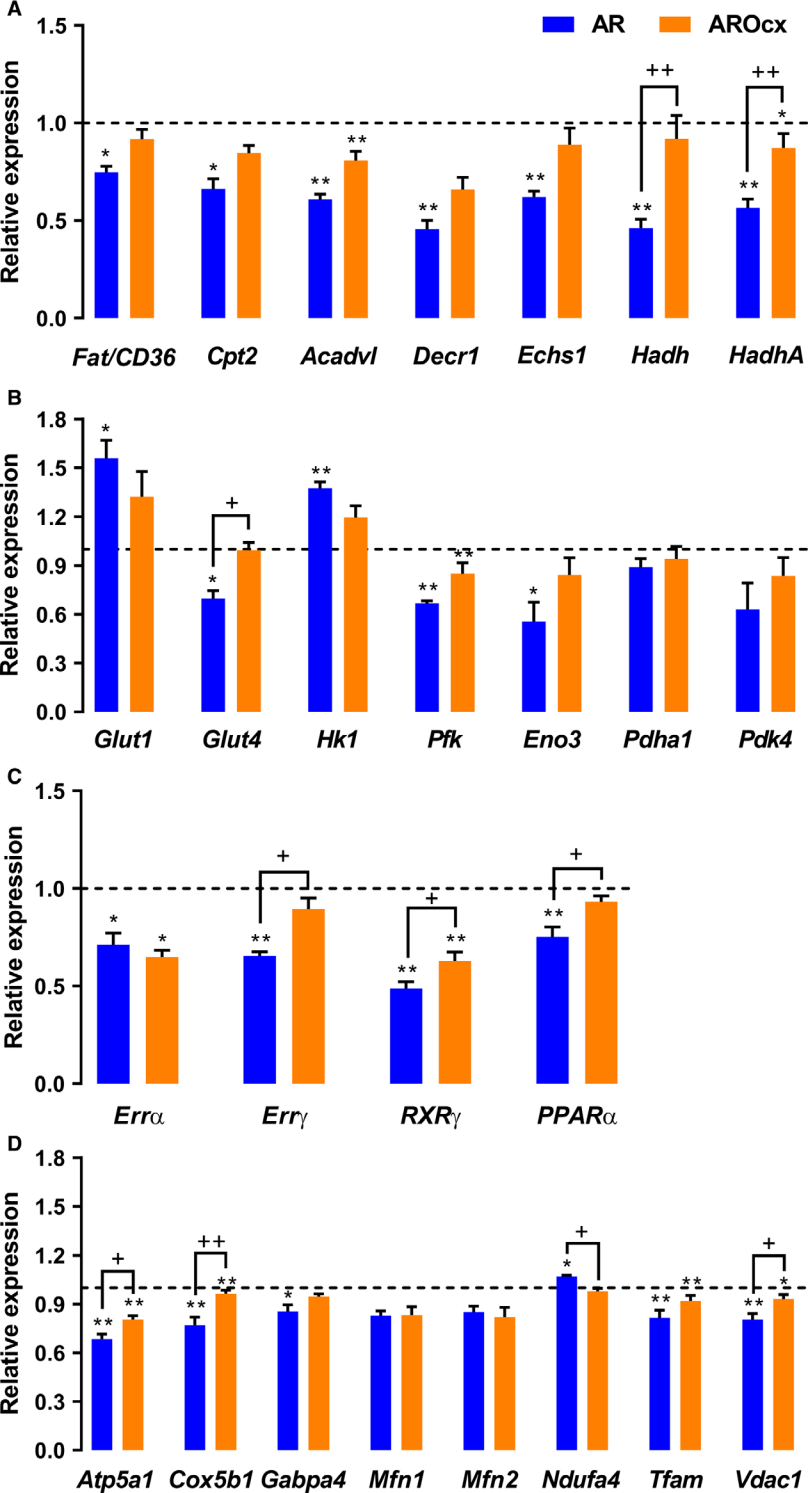
Evaluation by real‐time quantitative RT‐PCR of the LV mRNA levels of genes encoding for proteins related to fatty acid uptake and oxidation (A), for proteins relevant to glucose metabolism (B) for transcription factors related to fatty acid metabolism (C) and mitochondrial function and biogenesis (D). The results are reported in arbitrary units (AU) as the mean ± SEM (*n* = 5–6/gr.). Messenger RNA levels of the S group were normalized to 1 and are represented by the dotted line. *P < 0.05 or **P < 0.01 versus their respective sham group and ^+^ *P* < 0.05 or ^++^ *P* < 0.01 between the indicated groups.

A metabolic transition from fatty acid to glucose substrate use is a feature of pathological CH (Sambandam et al. 2002). Globally, mRNA levels of genes encoding fatty acid transporter (FAT) and enzymes implicated in fatty acid oxidation (FAO) were significantly lowered in AR animals, whereas only a few of these FAO genes were significantly downregulated in AROcx (Fig. 4A). There was no significant difference between the two sham groups (Fig. S2).

AR and Ocx (Fig. 4B) also influenced glucose metabolism and especially glycolysis. *Glut1* gene was significantly more expressed in the AR group whereas, *Glut4*, was significantly less expressed in AR animals and stayed at basal levels in AROcx. *Hk1* gene expression was significantly increased in AR animals. However, *Pfk* and *Eno3* mRNA levels, two other glycolytic enzymes, were significantly reduced in the AR group. *Pfk* was also reduced in the AROcx. There was no significant difference in *Pdha1* or *Pdk4* gene expression.

We then measured gene expression for *Errα*,* Errγ*,* Rxrγ*, and *Pparα*, transcription factors and cofactors involved in the regulation in myocardial energy metabolism. *Errα* and *Rxrγ* gene expression was decreased with AR, but *Errγ* and *Pparα* were only downregulated in intact AR rats (Fig. 4C). *Rxrγ* had a lower basal level in Ocx animals (Fig. S2), but the decrease in the AR group was still more important than in AROcx.

Metabolic changes are often accompanied by modifications in the expression of mitochondrial biogenesis and function genes (Fig. 4D). There was a downregulation of several of those genes in the AR group, except for mitofusin genes (*Mfn1* and *Mfn2*) that only tended to be reduced compared to sham rats. This downregulation was also present in the AROcx animals when compared to their respective controls. However, no significant difference was observed for the AROcx group for *Gabpa* and *Nduf4* genes. Nduf4 encodes for a subunit of the first complex of the respiratory chain. *Cox5b1*, which encodes for a subunit of cytochrome c (the fourth complex of the respiratory chain), was significantly more expressed in SOcx rats compared to S (Fig. S3).

**Figure 5:**
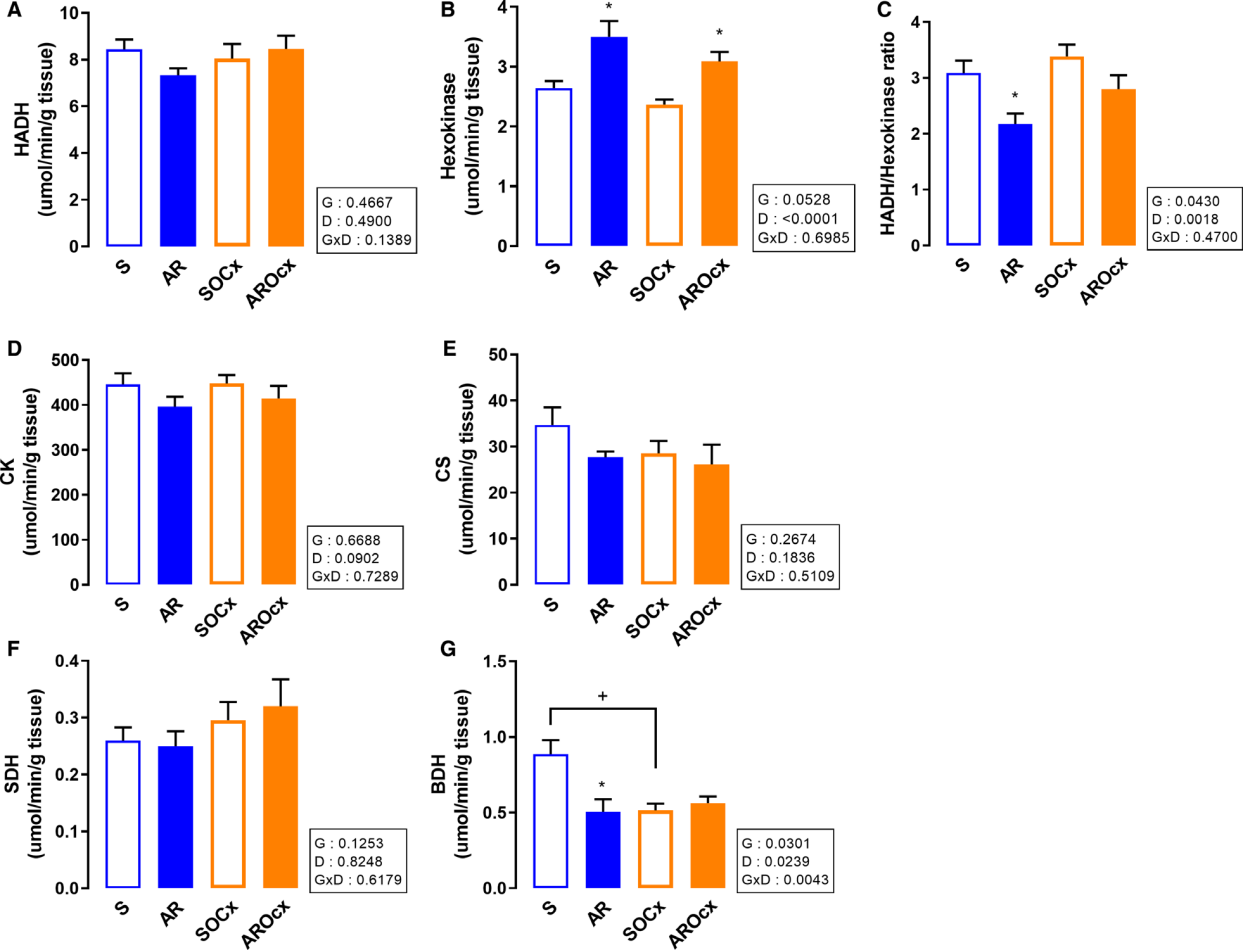
LV myocardial activity levels of enzymes implicated in *β*‐oxidation (HADH) and glycolysis (hexokinase). Hydroxyacyl‐Coenzyme A dehydrogenase (HADH) (A), hexokinase (B), the HADH/hexokinase ratio (C) creatine kinase (CK) (D), citrate synthase (CS) (E), succinate dehydrogenase (SDH) (F) and beta‐hydroxybutyrate dehydrogenase (BDH) (G). The results are reported as the mean ± SEM (*n* = 8/gr.). Probability values in the boxes are from a two‐way ANOVA and symbols, from Holm‐Sidak multiple comparisons post‐test. *P < 0.05 versus their respective sham group and ^+^ *P* < 0.05 between the indicated groups.

The enzymatic activity of the hydroxyacyl‐coenzyme A dehydrogenase (HADH), an enzyme implicated in the oxidation of medium chain fatty acid, was not significantly different between the groups although it tended be lowered in the AR group (Fig. 5A).

The hexokinase (HK) enzymatic activity determination was performed to measure myocardial glucose use capacity. HK activity was increased in both AR groups (Fig. 5B). Moreover, the HADH/HK activities ratio was significantly reduced in AR animals (Fig. 5C).

Nevertheless, the activity levels of other enzymes relevant for energy metabolism were not changed by either Ocx or AR. Creatine kinase (CK) activity was not different between the groups, except for AR, which tended to decrease it (Fig. 5D). AR or testosterone deficiency did not influence citrate synthase (CS) activity (Fig. 5E). Likewise, there was no significant change in succinate dehydrogenase (SDH) activity, an enzyme of both the citric acid cycle and the second complex of the respiratory chain (Fig. 5F). We then studied *β*‐hydroxybutyrate dehydrogenase (BDH) activity in order to measure ketone bodies use in the hypertrophied myocardium. AR reduced BDH activity in intact animals only. Ocx also decreased BDH activity in both sham and AR rats (Fig. 5G).

### Cell signaling

**Figure 6:**
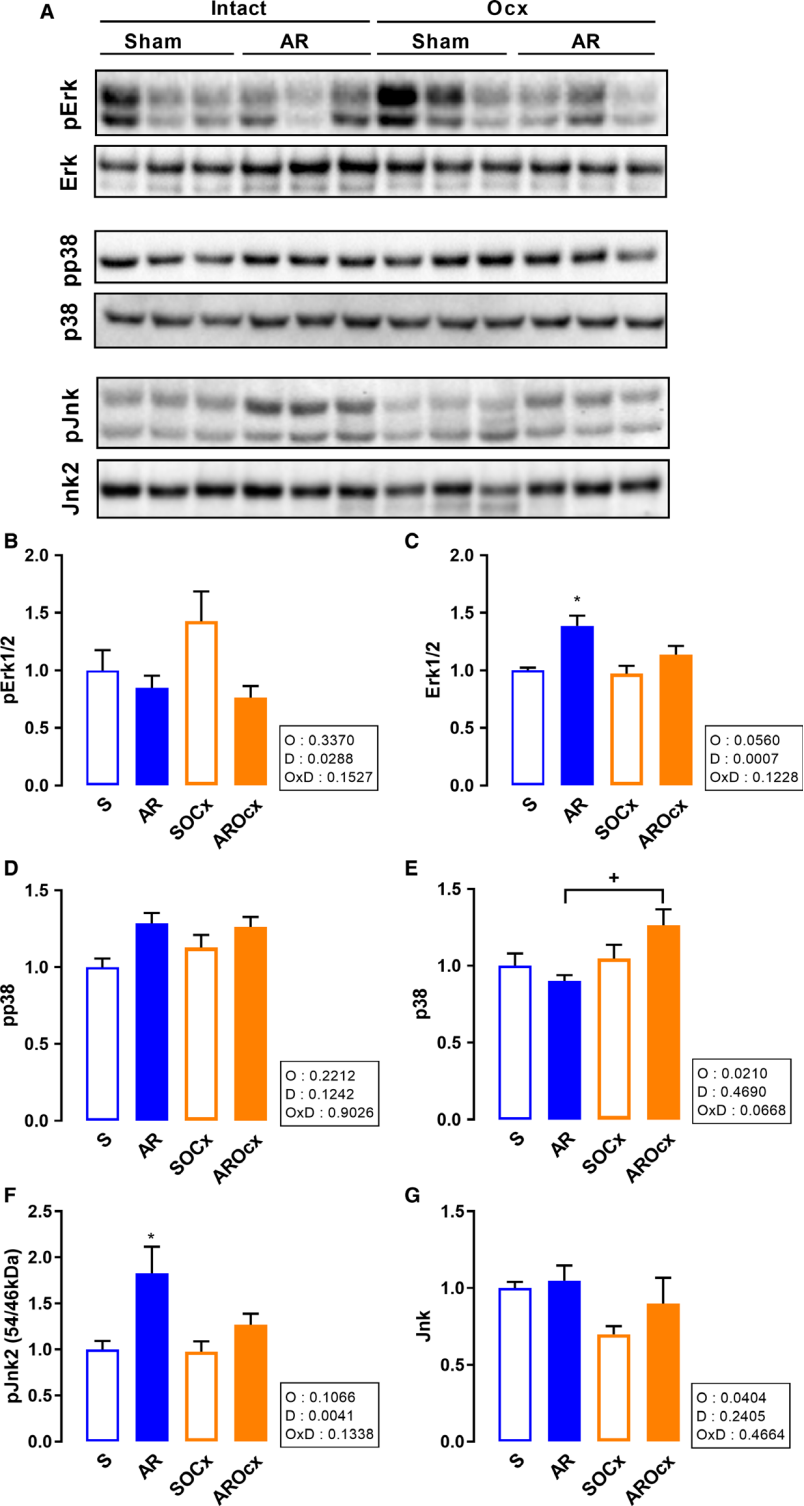
LV protein contents of Erk1/2, p38 and Jnk. Representative blots for each signaling molecules (A). Graphs B to G represent densitometric measurements after normalization. Phosphorylated Erk1/2 (B), total Erk1/2 (C), phosphorylated p38 (D), total p38 (E), phosphorylated Jnk2 (F), and total Jnk (G). The results are reported in arbitrary units (AU) as the mean ± SEM (*n* = 6/gr.). *P*‐values in the boxes are from a two‐way ANOVA and symbols, from Holm‐Sidak multiple comparisons post‐test. *P < 0.05 versus their respective sham group and ^+^ *P* < 0.05 between the indicated groups.

Mitogen‐activated protein kinases (MAPK) pathways are implicated in cell growth, cardiac myocytes hypertrophy, and response to stress (Heineke and Molkentin 2006). Phospho‐Erk1/2 content decreased in AR groups, this more markedly in Ocx animals, whereas total Erk1/2 significantly increased (Fig. 6A–C). Phospho‐p38 contents were mostly stable between groups. There was an increase in total p38 content in the LV of Ocx rats, (Fig. 6A, D and E). Phospho‐Jnk2 levels increased in AR groups. This increase was more important in the intact animals. There was no significant difference between the Ocx groups. Finally, total Jnk2 was reduced in Ocx groups (Fig. 6A, F and G).

**Figure 7:**
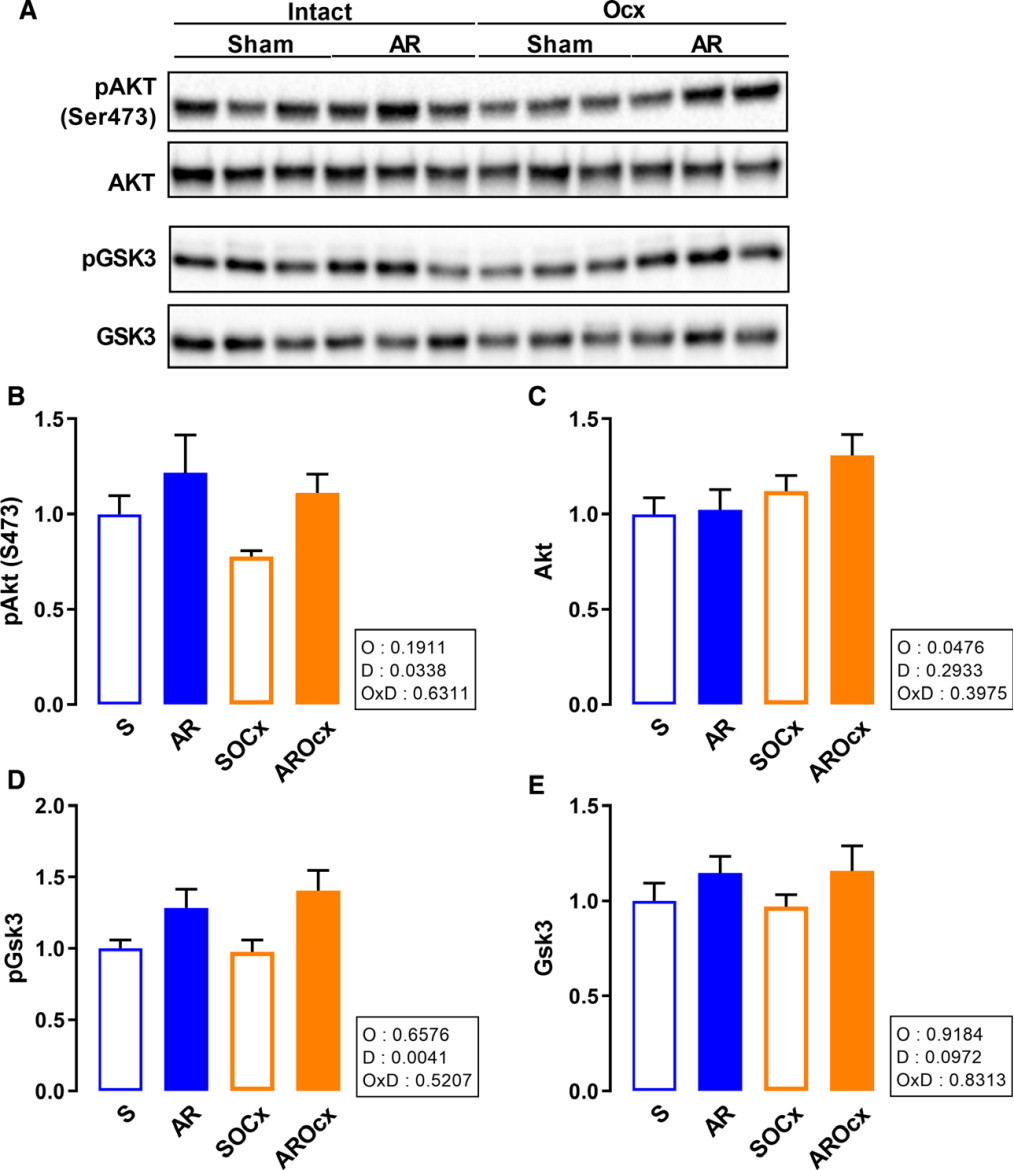
LV protein content of Akt and Gsk3. Representative blots for each signaling molecules (A). Graphs B to E represent densitometric measurement after normalization. Phosphorylated Akt on serine 473 (B), total Akt (C), phosphorylated Gsk3 (D), total Gsk3 (E). The results are reported in arbitrary units (AU) as the mean ± SEM (*n* = 6/gr.). Probability values in the boxes are from a two‐way ANOVA and symbols, from Holm‐Sidak multiple comparisons post‐test. *P < 0.05 versus their respective sham group and ^+^ *P* < 0.05 between the indicated groups.

Protein kinase B (Akt) is implicated in both hypertrophic and survival signaling pathways (Heineke and Molkentin 2006). Phospho‐Akt (S473) levels were significantly increased in AR groups and most clearly in Ocx rats (Fig. 7A–C). AR also increased levels of the phosphorylated and inactive form of Gsk3 (Fig. 7A, D and E).

S6, a ribosomal protein downstream of Akt and the mTORC1 (mTOR complex 1) and implicated in the regulation of protein synthesis and cell growth, was then analyzed. Phospho‐S6 was decreased by Ocx, but total form levels were constant between the groups (Fig. S4A–C). Loss of androgens significantly also decreased phosphorylated and total focal adhesion kinase (Fak) contents whereas AR had no effect (Fig. S4A, D and E). Finally, protein kinase D (Pkd) phosphorylated form was also decreased by Ocx, but did not change with AR. Total Pkd increased with the loss of androgens (Fig. S4A, F and G).

## Discussion

Our results suggest that androgens are important drivers of pathological cardiac hypertrophy development caused by severe left ventricle overload from chronic aortic regurgitation. Testosterone deficiency in AR rats resulted in reduced cardiac hypertrophy, less LV dilation, better function and a strong tendency for better survival of the animals. Changes in LV expression for an important number of genes associated with pathological myocardial remodeling were less marked in AROcx animals than in intact AR. In fact, the LV gene expression pattern we observed in AROcx rats had similarities with the one we reported recently in a study comparing AR females to males (Beaumont et al. 2017). Surprisingly, AR females had a stronger hypertrophic response to LV VO from AR than males, the opposite we observed with AROcx males. This suggests that LV hypertrophy and the LV transcriptional response may not be completely linked together and that other factors are implicated.

Orchiectomy reduced general growth of animals, which may be explained by the loss of androgens and their anabolic effects (Kloner et al. 2016). In parallel, normal cardiac growth was also reduced in Ocx animals. Loss of androgens was associated with a decrease in the basal activation levels of several signaling molecules such as S6 kinase, FAK, and PKD (PKC*μ*). Physiological heart growth and maladaptive hypertrophy development share and have distinctive signaling pathways. Activation of the Akt/mTOR pathway is associated with pathological CH (even in AR) although normal cardiac growth also relies upon this pathway (Ha et al. 2005; Kemi et al. 2008; Maillet et al. 2013; Volkers et al. 2013; Drolet et al. 2015). We tried, in this study, to better identify the contribution of androgens to pathological CH. This is why comparisons of AR and AROcx groups were mostly made versus their respective sham‐operated counterparts. Obviously, we cannot totally exclude that inhibition of normal cardiac growth by androgen removal may have also reduced the extent of the hypertrophic response to a similar volume overload at the start of the protocol in Ocx rats. This reduced hypertrophic response is accompanied by an attenuated LV gene expression response to AR. Moreover, Ocx had only little effects on basal LV gene expression in sham animals suggesting that changes in AR animals were related to the response to the overload and not to castration.

Even if VO severity was similar between both AR groups, stress and hypertrophic markers measured in this study pointed out that non‐Ocx animals myocardium was more responsive to the hemodynamic stress than those deprived of androgens. For instance, LV pJnk content was increased in both AR groups but this was more important in intact animals. Jnk can be activated by stress stimuli and its differential activation could explain in part differences in the cardiac response to overload between AR and AROcx rats (Sabapathy et al. 2004).

Hypertrophic markers gene expression results showed that the myocardium of AR animals responded to the increased LV wall stress, as expected. Interestingly, some of those genes had lower mRNA levels in the LV of AROcx rats (*Anp* and *Trpc6*). This suggests that androgens may be implicated in the myocardial response to mechanical wall stress. Interestingly, FAK (focal adhesion kinase), a mediator of integrin activation, is strongly downregulated in Ocx myocardium regardless of the presence or not of a hemodynamic stress.

The effects of testosterone deficiency did not seem to involve Akt, Gsk3, or p38 signaling. All AR animals (Ocx or not) had higher levels of pAkt and thus seemed to have enhanced Akt activity, as the pGsk3 content was similarly increased. Androgens action can be mediated by delayed genomic effect (classic androgen receptor) and a rapid non‐genomic effect via G‐coupled receptors such as GPCR6A or ZIP9. Testosterone has been shown to be pro‐hypertrophic in cultured neonatal rat cardiac myocytes (Marsh et al. 1998; Vicencio et al. 2006; Altamirano et al. 2009) mostly, but non only, via genomic or DNA‐binding effects. It is believed that testosterone leads probably via Akt to the phosphorylation and inhibition of Gsk3, which is considered an anti‐hypertrophic factor in cardiac cells (Duran et al. 2016; Lucas‐Herald et al. 2017). Our observation seems to be somewhat in contradiction with previous reports of the implication of this pathway to explain hypertrophic actions of androgens. We observed activation of Akt in AR rats and increased phosphorylation of Gsk3 but loss of testosterone had no effects on these parameters. The study of the mechanisms implicated in testosterone hypertrophic effects was mostly studied in cultured cardiac myocytes and not in living models of hypertrophy. Testosterone has also been shown to cause a raise in cytosolic calcium levels leading to the activation of calcineurin and NFAT, which are implicated in maladaptive hypertrophy development (Duran et al. 2016). The increased pJnk content we observed in the intact AR group could antagonize calcineurin effects through phosphorylation of NFAT. However, Jnk is not able to totally block calcineurin hypertrophic effects, which maintains the balance toward hypertrophic stimulation (Liang et al. 2003). In addition, testosterone stimulation of cardiomyocytes can result in S6 phosphorylation, which mediate cardiac growth and/or hypertrophy (Altamirano et al. 2009).

As we had previously reported in this rat model of LV volume overload, many genes related to myocardial energy metabolism were modulated (Arsenault et al. 2013; Roussel et al. 2015; Beaumont et al. 2017). As observed for females compared to intact AR males, AROcx rats displayed less modulation of those genes (Beaumont et al. 2017). This was also true for several genes implicated in the mitochondrial function. The expected shift toward glucose utilization from fatty acids by the myocardium was observed in intact AR rats where hexokinase activity was enhanced and a decrease in HADH/HK ratio was recorded. There was no change in activities of other energy metabolism enzymes studied (i.e., citrate synthase, succinate dehydrogenase and creatine kinase) suggesting that only some of these enzymes are influenced by either AR or hormonal status at this stage of the disease. Moreover, ketone body metabolism also seems impaired by Ocx and AR. Conversely, ketone body use usually increases in heart hypertrophy (pressure overload models) and HF (Aubert et al. 2016). Pathological remodeling of the heart frequently involves downregulation of fatty acids use as energy substrates in order to decrease oxygen consumption (Sambandam et al. 2002; Roussel et al. 2015). Here, we show that hormonal status of AR males influences their myocardial metabolic profile. In addition, estrogens are not the only mediator of sexual dimorphism in the metabolic profile induced by heart hypertrophy, as it has been proposed (Peterson et al. 2007). Androgen deprivation by Ocx can reduce metabolic changes. Our results suggest that AROcx animals may have a relatively preserved myocardial energy metabolism, which could help to respond to the higher energy demands from hypertrophy by preserving fatty acids use. This also suggests that androgens may contribute to the myocardial energy switch usually observed during pathological hypertrophy. The role of androgens on heart energy metabolism has not received a lot of attention in the past. However, testosterone has been shown to increase glucose use by cardiac myocytes in culture by increasing Glut‐4‐mediated glucose entry (Wilson et al. 2013).

There is no clear evidence of androgens effects on the hypertrophic heart and on HF in the literature. HF male patients often display hypoandrogenism. Androgens therapy in HF patients sometimes helps maintaining functional capacity, but these benefits are probably mediated via the peripheral effects of androgens on skeletal muscle, without directly improving heart function (Toma et al. 2012). In addition, different types of androgens may not have the same impact on the heart. Testosterone stimulates the coronary vasodilatation (Webb et al. 1999), whereas dihydrotestosterone (DHT, the product of testosterone transformation by the 5*α*‐reductase) impairs heart function and adaptation to stress by enhancing fibrosis (Montalvo et al. 2012). Moreover, even if both testosterone and DHT induce hypertrophy in cardiomyocytes, only DHT increases *Anp* levels (Marsh et al. 1998). In fact, 5*α*‐reductase activity (responsible for conversion of testosterone to DHT) and DHT levels are increased in the hypertrophic heart, which could enhance DHT negative effects (Thum and Borlak 2002; Zwadlo and Borlak 2012).

One limitation of our study is that we did not study the effect of androgen replacement therapy on Ocx animals. It is not clear if we could have reproduced physiological androgen levels in our animals over a long period of 6 months as well as the natural balance between testosterone and DHT. Also, these levels would have to be monitored to avoid supra‐physiological levels of androgens, which are known to be deleterious (Pirompol et al. 2016). Another limitation is that rats were orchiectomized at a relatively young age (8 weeks), which may have influenced the heart response to the hemodynamic stress. Male rats continue to grow and gain weight until late in their first year of life (Plante et al. 2008; Lachance et al. 2009a). Experiments in older rats (>12 months of age) could have been informative and should be performed in the future. Unlike the situation encountered for most human patients, AR was induced acutely in the animals enacting a probably more intense response of the heart than the one experienced in patients.

In conclusion, we showed that androgens reduce the hypertrophic response of the heart caused by severe and chronic volume overload from aortic valve regurgitation. Androgens deficiency seems to reduce the level of response of the myocardium to an increased mechanical stress probably resulting in less alterations in LV gene expression, extracellular matrix remodeling, and maintained normal energetics. This has similarities with the situation we observed in AR females (Beaumont et al. 2017). Unlike AR females though, Ocx males have reduced hypertrophy suggesting that other factors probably related to biological sex and/or estrogens have to be taken into consideration to explain this sexual dimorphism. Estrogens have received more attention in the study of pre‐clinical models of cardiac hypertrophy. Here, we show that androgens have also a major effect in mediating sexual dimorphism in the heart especially in the response to an overload.

## Conflict of Interest

The authors have no conflict of interest to declare.

## Supporting information




**Table S1.** Name and abbreviation of all primers used for gene expression analysis by quantitative RT‐PCR.
**Figure S1**. Comparison of SOcx with S with real‐time quantitative RT‐PCR of the LV mRNA levels of genes encoding for hypertrophy (A) extracellular matrix remodeling (B) markers.
**Figure S2**. Comparison of SOcx with S with real‐time quantitative RT‐PCR of the LV mRNA levels of genes encoding for glucose uptake and glycolysis (A), fatty acid oxidation (B), and transcription factor (C) markers.
**Figure S3**. Comparison of SOcx with S with real‐time quantitative RT‐PCR of the LV mRNA levels of genes encoding for mitochondrial function markers.
**Figure S4**. LV protein contents of S6, Fak, and Pkd. Representative blots for each signaling molecules (A).
