# Supplementary material for: Testosterone deficiency reduces cardiac hypertrophy in a rat model of severe volume overload
Source: Physiol Rep. 2019 May 3;7(9):e14088. doi: 10.14814/phy2.14088 (PMC6499867; doi:10.14814/phy2.14088)
Supplement: Supplementary file 1 — Table S1. Name and abbreviation of all primers used for gene expression analysis by quantitative RT‐PCR. Figure S1. Comparison of SOcx with S with real‐time quantitative RT‐PCR of the LV mRNA levels of genes encoding for hypertrophy (A) extracellular matrix remodeling (B) markers. Figure S2. Comparison of SOcx with S with real‐time quantitative RT‐PCR of the LV mRNA levels of genes encoding for glucose uptake and glycolysis (A), fatty acid oxidation (B), and transcription factor (C) markers. Figure S3. Comparison of SOcx with S with real‐time quantitative RT‐PCR of the LV mRNA levels of genes encoding for mitochondrial function markers. Figure S4. LV protein contents of S6, Fak, and Pkd. Representative blots for each signaling molecules (A). [file PHY2-7-e14088-s001.pdf]

Supplemental tables and figures

Table S1. Name and abbreviation of all primers used for gene expression analysis by quantitative RT-PCR. The table also includes catalogue numbers (from IDT or Qiagen) and the amplicon of the primers

mARN	*Symbol*	Cat. No.	Amplicon (pb)
acetyl-CoA acyltransferase 2	*Acaa2*	Rn.PT.58.5300756	111
acyl-CoA dehydrogenase, very long chain	*Acadvl*	Rn.PT.58.13279450	147
acetyl CoA acetyltransferase 1	*Acat*	Rn.PT.58.18447027	102
ANT-1 solute carrier family 25, member 4	*Ant1, Slc25a4*	Rn.PT.58.36556272	103
ATP synthase, H+ transporting, mitochondrial F1 complex, alpha subunit 1, cardiac muscle	*Atp5α1*	Rn.PT.58.6992257	100
CD36/ fatty acid translocase	*Fat/CD36*	Rn.PT.58.13645667	122
procollagen-1 alpha-1	*Col1α1*	Rn.PT.58.7562513	134
procollagen-3 alpha-1	*Col3α1*	Rn.PT.58.11138874	100
cytochrome c oxidase subunit 5B	*Cox5b1*	Rn.PT.58.8467954	138
cartinine palmitoyltransferase 1b, muscle	*Cpt1b*	Rn.PT.58.9348596	105
carnitine palmitoyltransferase 2	*Cpt2*	Rn.PT.58.5579430	107
carnitine O-acetyltransferase	*Crat*	Rn.PT.58.36282119	97

connective tissue growth factor	*Ctgf*	QT00182021	102
2,4-dienoyl CoA reductase 1, mitochondrial	*Decr1*	Rn.PT.58.44352482	120
enoyl CoA hydratase 1, peroxisomal	*Ech1*	Rn.PT.58.33832465	99
enoyl CoA hydratase, short chain 1, mitochondrial	*Echs1*	Rn.PT.58.44352482	95
enoyl CoA delta isomerase 1	*Eci*	Rn.PT.58.37662439	119
enolase 3, beta	*Eno3*	Rn.PT.58.37792224	103
estrogen related receptor, alpha	*Errα*	Rn.PT.58.5170310	111
estrogen related receptor, gamma	*Errγ*	Rn.PT.58.8028733	141
osteosarcoma viral oncogene homolog	*Fos*	QT01576330	73
Fibronectin 1	*Fn1*	Rn.PT.58.18226984	114
GA binding protein transcription factor, alpha	*Gapba4*	Rn.PT.58.12555216	137
solute carrier family 2 member 1	*Glut1, Slc2a1*	QT00178024	85
solute carrier family 2 member 4	*Glut4, Slc2a4*	Rn.PT.58.34939400	115
hydroxyacyl-CoA dehydrogenase	*Hadh*	Rn.PT.58.17867024	135
hydroxyacyl-CoA dehydrogenase alpha	*HadhA*	Rn.PT.58.46222281	138
hydroxyacyl-CoA dehydrogenase beta	*HadhB*	Rn.PT.58.7613498	130
Hexokinase 1	*Hk1*	Rn.PT.58.8913174	108

Hexokinase 2	*Hk2*	Rn.PT.58.46137159	110
Krüppel-like factor 15	*Klf15*	Rn.PT.58.12431283	129
Lysyl oxidase	*Lox1*	Rn.PT.58.10677971	150
mitofusin 1	*Mfn1*	Rn.PT.58.44207597	106
mitofusin 2	*Mfn2*	Rn.PT.58.13375660	124
matrix metalloproteinase-2	*Mmp2*	Rn.PT.58.44737355	87
myosin, heavy polypeptide 6, cardiac	*Myh6*	Rn.PT.58.8646063	150
myosin, heavy polypeptide 7, cardiac	*Myh7*	Rn.PT.58.34623828	125
NADH: ubiquinone oxidoreductase subunit A4	*Ndufa4*	Rn.PT.58.11318539	139
natriuretic peptide precursor type A	*Nppa, Anp*	Rn.PT.58.5865224	79
natriuretic peptide precursor type B	*Nppb, Bnp*	Rn.PT.58.5595685	108
pyruvate dehydrogenase alpha 1	*Pdha1*	Rn.PT.58.14147312	145
pyruvate dehydrogenase kinase, isozyme 4	*Pdk4*	Rn.PT.58.6203434	135
Phosphofructokinase	*Pfk*	Rn.PT.58.17873275	122
Peroxisome proliferator-activated receptor alpha	*Pparα*	Rn.PT.58.35488724	134
cyclophilin A	*Ppia*	Rn.PT.39a,22214830	140
retinoid X receptor gamma	*Rxrγ*	Rn.PT.58.6519292	103
transcription factor A, mitochondrial	*Tfam*	Rn.PT.58.13772978	124
Tissue inhibitor of metalloproteinases 1	*Timp1*	Rn.PT.58.34442920	127

transient receptor potential cation channel, subfamily C, member 6	*Trpc6*	Rn.PT.58.18089975	94
voltage-dependent anion channel 1	*Vdac1*	Rn.PT.58.37188705	116

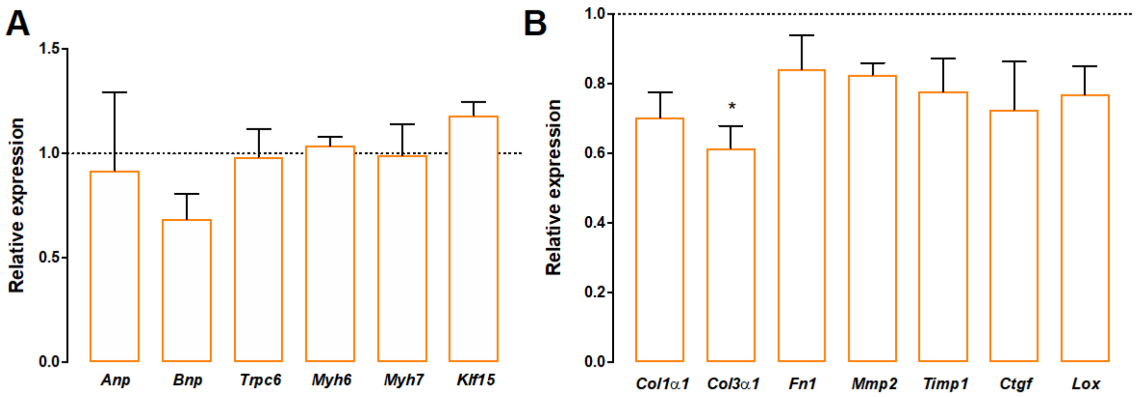

Figure S1. Comparison of SOcx with S with real-time quantitative RT-PCR of the LV mRNA levels of genes encoding for hypertrophy (A) extracellular matrix remodeling (B) markers. The mRNA levels of the S group were normalized to 1 and are represented by the dotted line. The results are reported in arbitrary units (AU) as the mean ± SEM (n=6/gr.). *p <0.05 vs. the S group.

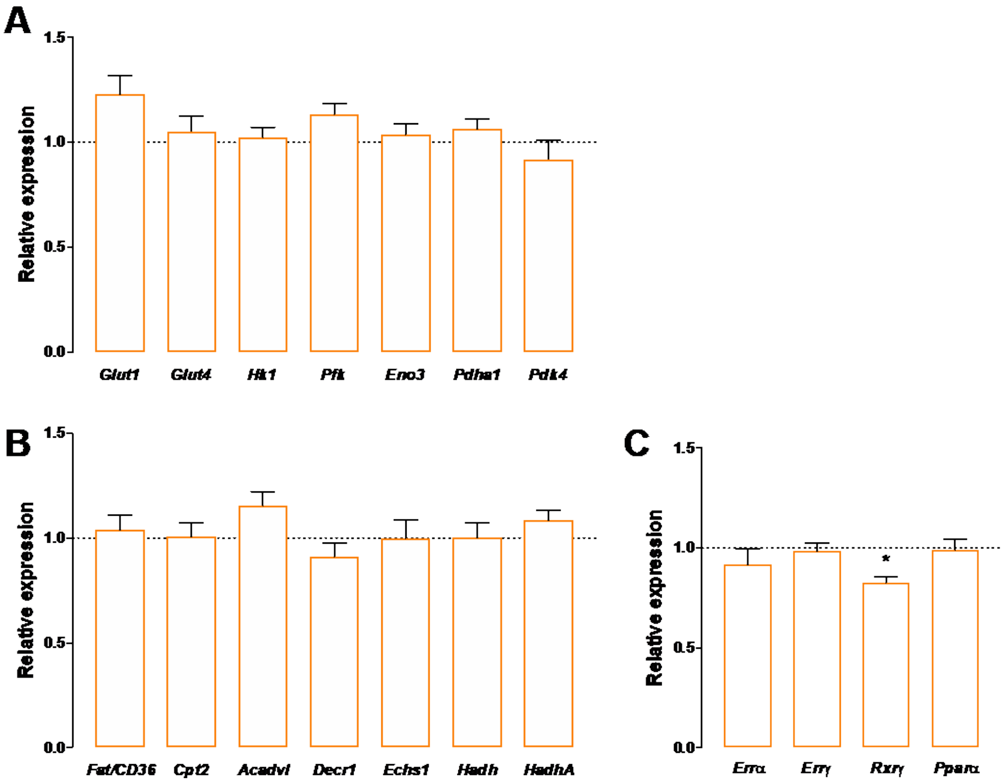

Figure S2. Comparison of SOcx with S with real-time quantitative RT-PCR of the LV mRNA levels of genes encoding for glucose uptake and glycolysis (A), fatty acid oxidation (B) and transcription factor (C) markers. The mRNA levels of the S group were normalized to 1 and are represented by the dotted line. The results are reported in arbitrary units (AU) as the mean ± SEM (n=6/gr.). *p<0.05 vs. the S group.

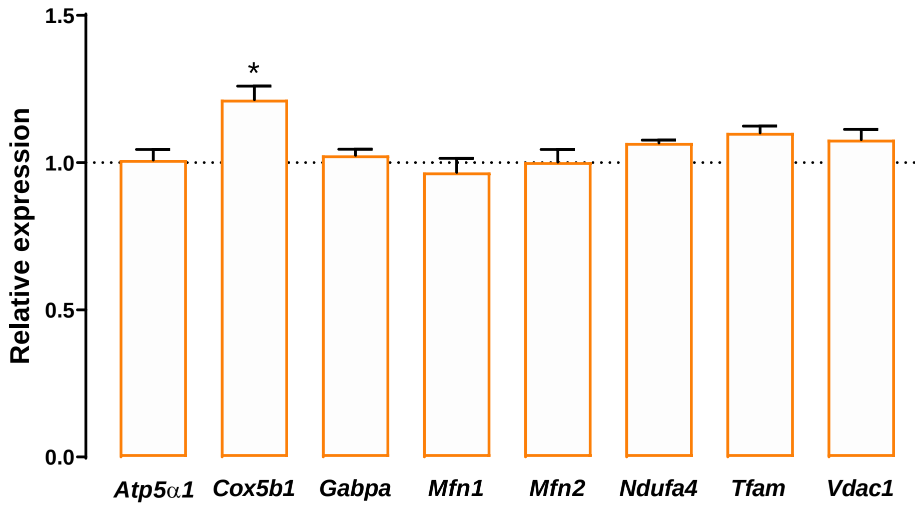

Figure S3. Comparison of SOcx with S with real-time quantitative RT-PCR of the LV mRNA levels of genes encoding for mitochondrial function markers. The mRNA levels of the S group were normalized to 1 and are represented by the dotted line. The results are reported in arbitrary units (AU) as the mean ± SEM (n=6/gr.). *p<0.05 vs. the S group.

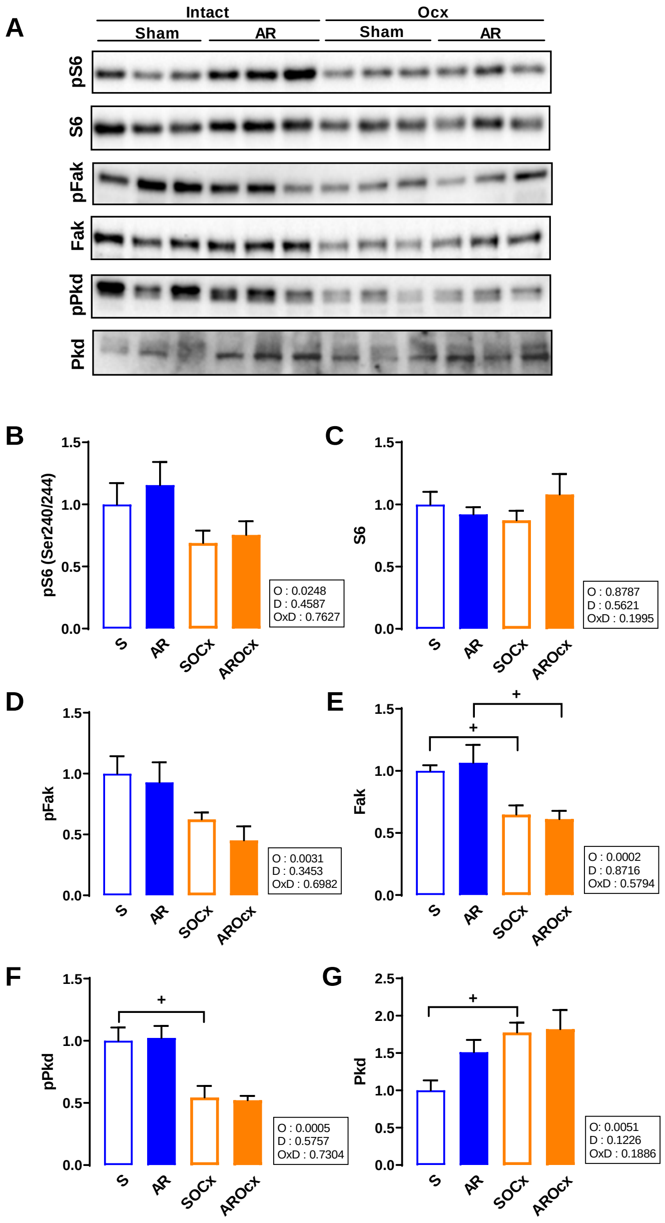

Figure S4. LV protein contents of S6, Fak and Pkd. Representative blots for each signaling molecules (A). Graphs B to G represent densitometric measurement after normalization. Phosphorylated S6 on serines 240 and 244 (B), total S6 (C), phosphorylated Fak (D), total Fak (E), phosphorylated Pkd (F) and total Pkd (G). The results are reported in arbitrary units (AU) as the mean ± SEM (n=6/gr.). Probability values in the boxes are from a two-way ANOVA and symbols, from Holm-Sidak multiple comparisons post-test. *: $p<0.05$ vs. their respective sham group and +: $p<0.05$ between the indicated groups.
